# Artificial intelligence solutions enabling sustainable agriculture: A bibliometric analysis

**DOI:** 10.1371/journal.pone.0268989

**Published:** 2022-06-09

**Authors:** Priya Rani Bhagat, Farheen Naz, Robert Magda

**Affiliations:** 1 Institute of Agricultural and Food Economics, Hungarian University of Agriculture and Life Sciences, Gödöllő, Hungary; 2 North-West University, Vanderbijlpark, South Africa; University of Bonab, ISLAMIC REPUBLIC OF IRAN

## Abstract

There is a dearth of literature that provides a bibliometric analysis concerning the role of Artificial Intelligence (AI) in sustainable agriculture therefore this study attempts to fill this research gap and provides evidence from the studies conducted between 2000–2021 in this field of research. The study is a systematic bibliographic analysis of the 465 previous articles and reviews done between 2000–2021 in relation to the utilization of AI in sustainable methods of agriculture. The results of the study have been visualized and presented using the VOSviewer and Biblioshiny visualizer software. The results obtained post analysis indicate that, the amount of academic works published in the field of AI’s role in enabling sustainable agriculture increased significantly from 2018. Therefore, there is conclusive evidence that the growth trajectory shows a significant climb upwards. Geographically analysed, the country collaboration network highlights that most number of studies in the realm of this study originate from China, USA, India, Iran, France. The co-author network analysis results represent that there are multi-disciplinary collaborations and interactions between prominent authors from United States of America, China, United Kingdom and Germany. The final framework provided from this bibliometric study will help future researchers identify the key areas of interest in research of AI and sustainable agriculture and narrow down on the countries where prominent academic work is published to explore co-authorship opportunities.

## 1. Introduction

As the world tries to battle through the novel challenges and obstacles caused by the Covid-19 pandemic and every sector faced detrimental impact of the pandemic at different stages of a business process. The agriculture sector is one of these sectors which faced several disruptions including labor supply for production, reduced transportation of agriculture produce, and risks associated with agriculture markets. Therefore, artificial intelligence (AI) systems and solutions will find contemporary applications in the field of agriculture and sustainable farming practices. Evidently, the pandemic is not a temporary disruption of normal but a milestone that will change the course of modern-history towards a sustainability driven society [1, 2]. Sustainability is a multidimensional discipline that has been the popular research interest of scholars for the past few decades [3, 4]. Because of its multidisciplinary attribute sustainability encompasses a wide variety of subjects ranging from climate, environment, green economy, food safety, sustainable agriculture, clean technologies etc. In a 2018 research article, Ávila et al. highlighted that in the field of education in sustainability, expansive research in every contributing domain is necessary to embark on the trail to global development [5].

The concept of “sustainable agriculture” has received increased attention in recent years with the rising technological improvements. In a 2020 keyword citation burst analysis on ecological modernization approach by Rocchi et al., “sustainable agriculture” consistently shows very high citation burst in the past and current literature [6]. Due to its high popularity among researchers the more recently published bibliometrics studies conduct in the realm of sustainable agriculture explore the interactions between sustainable agri-food systems with the economy, society, and policy making [7], agriculture systems modernization approach [6], big data in sustainable agriculture [8]. In terms of Artificial Intelligence in agriculture, recent articles provide bibliometric analysis of the crossover between remote sensing technologies and agriculture [9], advanced information and communication technology in agriculture [10], global trends in precision agriculture technology [11].

In the scope of this review, sustainable agriculture is defined as, the agricultural practices that ensure fulfillment of present day and future food and nutrition requirements of the society, while maximizing the net advantage towards the ecosystem, society, and earth when all its implication on costs and benefits are monitored [12]. Artificial intelligence (AI) was first coined by John McCarthy in 1956 and then many different definitions arise over the years but in the scope of this study, it is defined in the rational approach as a system that automates intelligent behaviour or acquires intelligence over time using computational programming and gives rational outputs to perform specific tasks without much human intervention [13].

The impact of the covid-19 pandemic on the agriculture sector is detrimental. There were various studies conducted in the past that observed the disruptions in the agriculture sector and provided sustainable solutions by employing AI technologies in creating sustainable agriculture. However, there is a dearth of studies that provided a state-of-the-art review of AI technologies application in sustainable agriculture. Therefore, this study was conducted to identify the current stage of knowledge concerning AI and sustainable agriculture and provided bibliometric and network analysis in this field. Also, this bibliometric research is motivated to bring together the highlights of the progress made in the application of AI in sustainable agriculture practices to inspire future applications of AI in this field. Hence, it is imperative for future researchers to reflect upon the past decade for academic literature in the field to be able to innovate according to the demands of the current pandemic situation [2]. This study attempts to compile, analyze, and identify the properties of the scholarly articles and reviews indexed in the Scopus database specifically for the keywords- artificial intelligence, Sustainable agriculture made available between the duration of 2000 to 2021. This study involves both quantifying and bringing out qualitative inferences from the size and features of previously published scholarly works from one or more source databases such as Scopus indexed database. The qualitative questions we intend to explore in the scope of this study are as follows:

1. What is the number of scientific articles and reviews published between the year 2000–2021 in the scope of AI and sustainable agriculture?
2. What is the growth trajectory in the number of works published in the area of AI and Sustainable agriculture?
3. What is the geographic distribution of the research work produced around the world in the field of study?
4. Which are the prominent journals and who are the eminent authors involved in the research of AI and sustainable agriculture?

Following this introduction, this article is structured in the following manner: in section 2 the materials and methods used are elaborated. Further, in section 2.1 the bibliometric analysis approach is explained, followed by section 3 which provides an overview and results, and finally, the article closes with sections 4, 5, and 6 providing a discussion about future research options, implications in the present and conclusion respectively.

## 2. Research methodology

The approach taken to conduct this study is a comprehensive bibliometric analysis of the previously published works of literature that incorporate the usage of AI in the field of sustainable agriculture between the duration of 2000 and 2021. A total of 637 articles were extracted from the Scopus database as a CVS file, out of which only 465 relevant journal articles and review papers were considered for maintaining the veracity of the resulting conclusions. Scopus as a source index, is highly regarded amongst academicians and researchers for searching legitimate scientific articles as it facilitates searching and extraction of specific keywords from the titles, citations, abstracts or keywords from the publications listed in the database [14].

Bibliometric analysis has been regarded as a reliable method to perform quantitative and empirical study and compilation of previously published works of literature in any field [15]. Pritchard in 1969 first vaguely defined bibliometrics as analyzing books, written documents, article or media communication by application of statistical and mathematical tools [16]. Broadus then in 1987 defined bibliometrics as “the quantitative study of physical published units, or of bibliographic units, or of the surrogates for either [17]". In the scope of this study it encompasses analysis methods such as citation network analysis, geographic network analysis, prominent countries and authors ranked and prominent word cloud. These tools provide a visual representation of the development of literature in the specified field over the course of time and highlight the impactful trending areas of research in the duration selected [18].

Visualization of the networks established from the large number of articles extracted is a key step in the bibliometric process which was done using multidimensional scaling there are various software tools available in the market like R package, iGraph package, VOSviewer and Biblioshiny [19]. Due to constant innovation and increased accessibility of advanced web-based and electronic bibliographic and referencing applications, the outputs from bibliometric analysis have greatly improved in quality [20]. Within the scope of this study, VOSviewer has been used to generate and visually represent the network of authors and countries as the VOSviewer platform provides the most suitable options for easily displaying the bibliometric maps and is easily understandable by any type of audience. Biblioshiny is used in the study to generate the prominent keyword cloud. It is an extension of the Bibliometrix R package web-interface used to visualize the clusters in the database.

### 2.1 Research method: Bibliometric study

**Fig 1:**
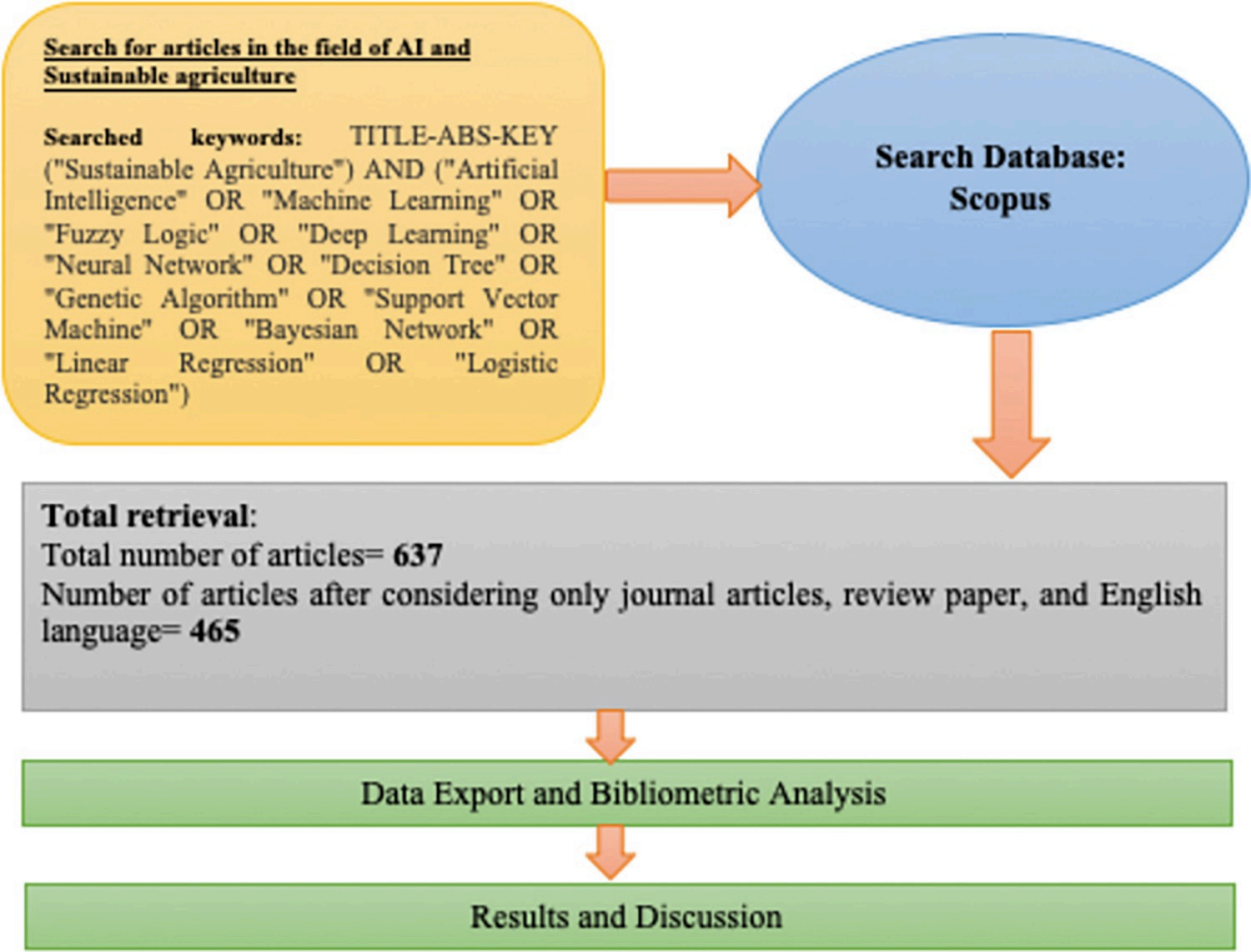
Bibliometric procedure used to conduct the study.

The procedure followed in this study was standard comprehensive bibliometric analysis which starts from keyword search in the Scopus search syntax as shown in the first step of Fig 1. The keywords indicate that articles and publications containing “Artificial Intelligence” or “Machine Learning” or “Robotics” etc. AND “Sustainable Agriculture” in their titles, keywords, or abstracts. The search was run, and the total number of articles obtained within the timespan of 2000 to 2021 and before any type of filtration was 637. The output was then refined for any redundancies. Thereafter, only journal articles, review papers, selecting only English papers, and within year 2000–2021 were considered for the study, bringing the total number of articles to 465.

This total consists of about 85.8% journal articles and 14.2% review papers. Further, about 46.6% of the publications were made during or after the onset of the Covid-19 pandemic. This set of indexes was then exported to Excel, VOSviewer, and Biblioshiny to obtain the network analysis, assessment of the growth trajectory, geographic distribution, identification of prominent authors, and keyword assessment. The ranking of country-wise publication of articles and citations were determined using Microsoft Excel features.

## 3. Results and overview

The Table 1 describes the important properties of the data set used for the study which helps in determining the overall scholarly or academic impact that has been made in the field of AI utilized in sustainable agriculture in the duration of 2000 to 2021. The total size of the literary works extracted for the study is 465. One of the most notable properties of this data set is that out of a total of 2005 authors who publish research in this field only 15 of them have produced single author documents. When compared to bibliometric analysis in other popular fields of academic study, these statistics have lower numbers which indicate that there is a need for more academic research and publication to provide knowledge and encourage the use of AI widely.

**Table 1:** Main information about literature in AI and sustainable agriculture.

Description	Results
**MAIN INFORMATION ABOUT DATA**	
Timespan	2000:2021
Sources (Journals, Books, etc.)	270
Documents	465
Average years from publication	3.68
Average citations per documents	17.92
Average citations per year per doc	3.37
References	33551
**DOCUMENT TYPES**	
articles	399
review	66
**DOCUMENT CONTENTS**	
	3288
Author’s Keywords (DE)	1787
**AUTHORS**	
Authors	2005
Author Appearances	2202
Authors of single-authored documents	15
Authors of multi-authored documents	1990
**AUTHORS COLLABORATION**	
Single-authored documents	16
Documents per Author	0.232
Authors per Document	4.31
Co-Authors per Documents	4.74
Collaboration Index	4.43

### 3.1 Year-wise article statistics

**Fig 2:**
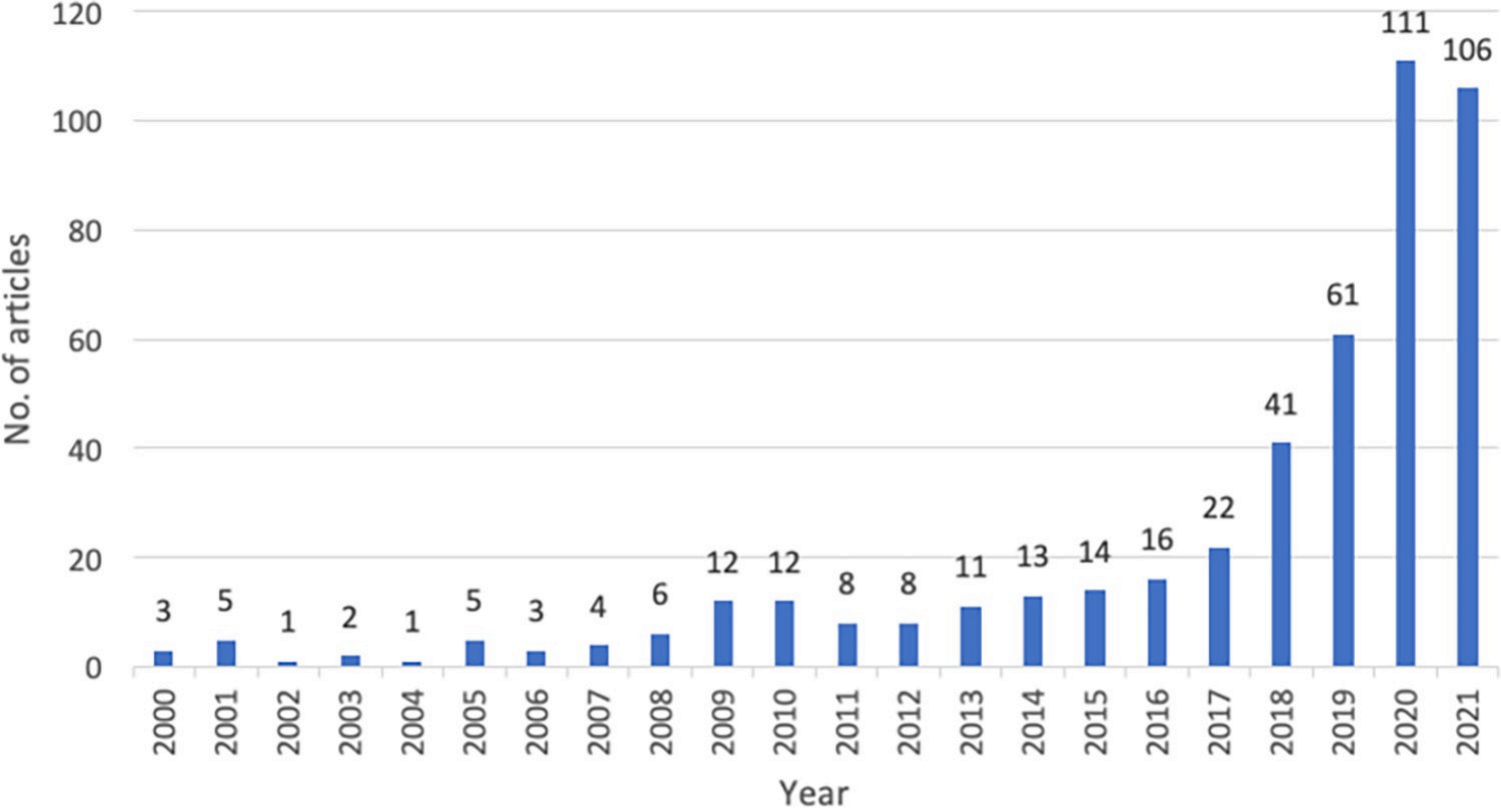
Year-wise articles.

Further, Fig 2 is used to visually depict the growth trajectory and shift of research interest in the use of AI in sustainable agriculture. The academic literature in this discipline had steady but moderate growth from the year 2000 to 2015. After which evidently, there was a greater impact Industry 4.0 had on sustainable agriculture practices and hence we can see the trend gradually increasing since 2017 and we see an approximately 255.7% increase in Scopus indexed articles and review published from 2019 to 2020 and mid 2021. This growth confirms a surge in scholarly inclination towards addressing the uses of AI in sustainable agriculture practices.

### 3.2 Country wise article statistics

In terms of the geographic distribution of the literary works, as per Table 2 about 54.8% of the publications are produced from China ranking it at number 1 out of top 20. The next countries leading knowledge production in use of AI in sustainable agriculture are USA, India, Iran, and France. The rest of the list also comprises of highly developed Anglo-American-European nations such as, Italy, UK, Germany, Spain, Australia, Netherlands, Turkey, Canada, Switzerland and Portugal, and other developing Asian economies such as Malaysia, Indonesia and Pakistan.

**Table 2:** Country wise production of articles.

No.	Region	Frequency	No.	Region	Frequency
**1**	China	210	**11**	Netherlands	37
**2**	USA	147	**12**	Brazil	33
**3**	India	102	**13**	Turkey	25
**4**	Iran	56	**14**	Malaysia	22
**5**	France	54	**15**	Canada	20
**6**	Italy	51	**16**	Indonesia	20
**7**	UK	48	**17**	Pakistan	18
**8**	Germany	46	**18**	Switzerland	18
**9**	Spain	43	**19**	Belgium	16
**10**	Australia	42	**20**	Portugal	16

### 3.3 Country wise citation statistics

The country wise citation ranking in Table 3, shows USA and China received a maximum number of citations 1938 and 1141, respectively. Then, the following countries with a considerable number of received citations are UK, Netherlands, and Germany. Whereas, Algeria shows the highest average article citation at 68.

**Table 3:** Country wise citation.

No.	Country	Total Citations	Ave. Article Citations	No.	Country	Total Citations	Ave. Article Citations
**1**	USA	1938	51	**11**	Iran	160	7.273
**2**	China	1141	17.554	**12**	France	151	12.583
**3**	UK	576	33.882	**13**	Algeria	136	68
**4**	Netherlands	453	41.182	**14**	Brazil	115	14.375
**5**	Germany	386	27.571	**15**	Canada	112	16
**6**	India	345	11.129	**16**	Sweden	112	112
**7**	Australia	343	31.182	**17**	Greece	96	16
**8**	Italy	286	15.889	**18**	Switzerland	93	15.5
**9**	Malaysia	249	31.125	**19**	Thailand	88	14.667
**10**	Spain	189	9.947	**20**	Austria	85	21.25

### 3.4 Ranking of key journals in the selected discipline

It is important for promising scholars to identify the role of prominent journals which are facilitating knowledge dissemination in the discipline of the use of AI in sustainable agriculture so that they can approach the industry leaders and foster further innovation and research potentials. In our analysis, we identified the top 20 journals that span into a wide variety of research disciplines (Table 4). To highlight a few, Sustainable practices (Sustainability), agricultural economy (Agronomy, Agronomy For Sustainable Development), technology in agriculture (Computers and Electronics in Agriculture-81 papers), environmental research (Science of the Total Environment, Journal of Cleaner Production, Agricultural Water Management), emerging technologies (Remote Sensing, Applied Sciences), Multidisciplinary (IEEE Access), environmental and sustainability studies (Land Use Policy), Environmental Monitoring and Assessment (Agriculture Ecosystems and Environment, Ecological Indicators) and the list includes furthermore areas. This indicates that there is a growing inclination towards cross-disciplinary exploration in attaining AI systems that can help make agriculture more sustainable in the future.

**Table 4:** Most relevant journals.

No.	Sources	Articles
**1**	Sustainability (Switzerland)	32
**2**	Agronomy	16
**3**	Computers And Electronics in Agriculture	12
**4**	Science of the Total Environment	12
**5**	Journal of Cleaner Production	9
**6**	Agricultural Water Management	7
**7**	Land Use Policy	7
**8**	Agriculture Ecosystems and Environment	6
**9**	Agronomy For Sustainable Development	6
**10**	Ecological Indicators	6

### 3.5 Ranking of key authors in the selected discipline

Using biblioshiny package in R-software, we have generated Table 5, which indicated the most prominent authors publishing in the domain of AI utilization in sustainable agriculture and farming. Based on the filtered database the papers, articles, and reviews are produced by 2005 authors in total. The top published authors come from China, USA, India, Iran and France based on their affiliate universities. The low number of articles even from the top authors suggest that this field of study is still in its emerging stages and increasing authorship and co-authorship has the potential of drawing further interest in this discipline.

**Table 5:** Most relevant authors.

No.	Authors	Articles
**1**	Li X	5
**2**	Wang X	5
**3**	Chen Z	4
**4**	Feng X	4
**5**	Kumar A	4
**6**	Kumar S	4
**7**	Liu Y	4
**8**	Wang Y	4
**9**	Zhang Y	4
**10**	Ahmad A	3

### 3.6 Ranking of key institutions in the selected discipline

The top ten affiliations by the number of articles produced are shown in Table 6. It is essential to determine which distinguished organizations and universities are leading the research scenario in the field of AI utilization in sustainable agriculture and related disciplines. According to the output from the analysis, Wageningen University, Netherlands has produced 13 scientific articles in the scope of the topic under study. The following China Agricultural University, and Northwest A&F University are from China, and each have published 12 and 10 scientific articles, respectively.

**Table 6:** Most important organizations.

No.	Affiliations	Articles
**1**	Wageningen University	13
**2**	China Agricultural University	12
**3**	Northwest A&F University	10
**4**	Michigan State University	9
**5**	University of Tehran	9
**6**	Sichuan Agricultural University	8
**7**	University of Bonn	8
**8**	Nanjing Agricultural University	7
**9**	University of California	7
**10**	Huazhong Agricultural University	6

### 3.7 Keyword analysis

**Fig 3:**
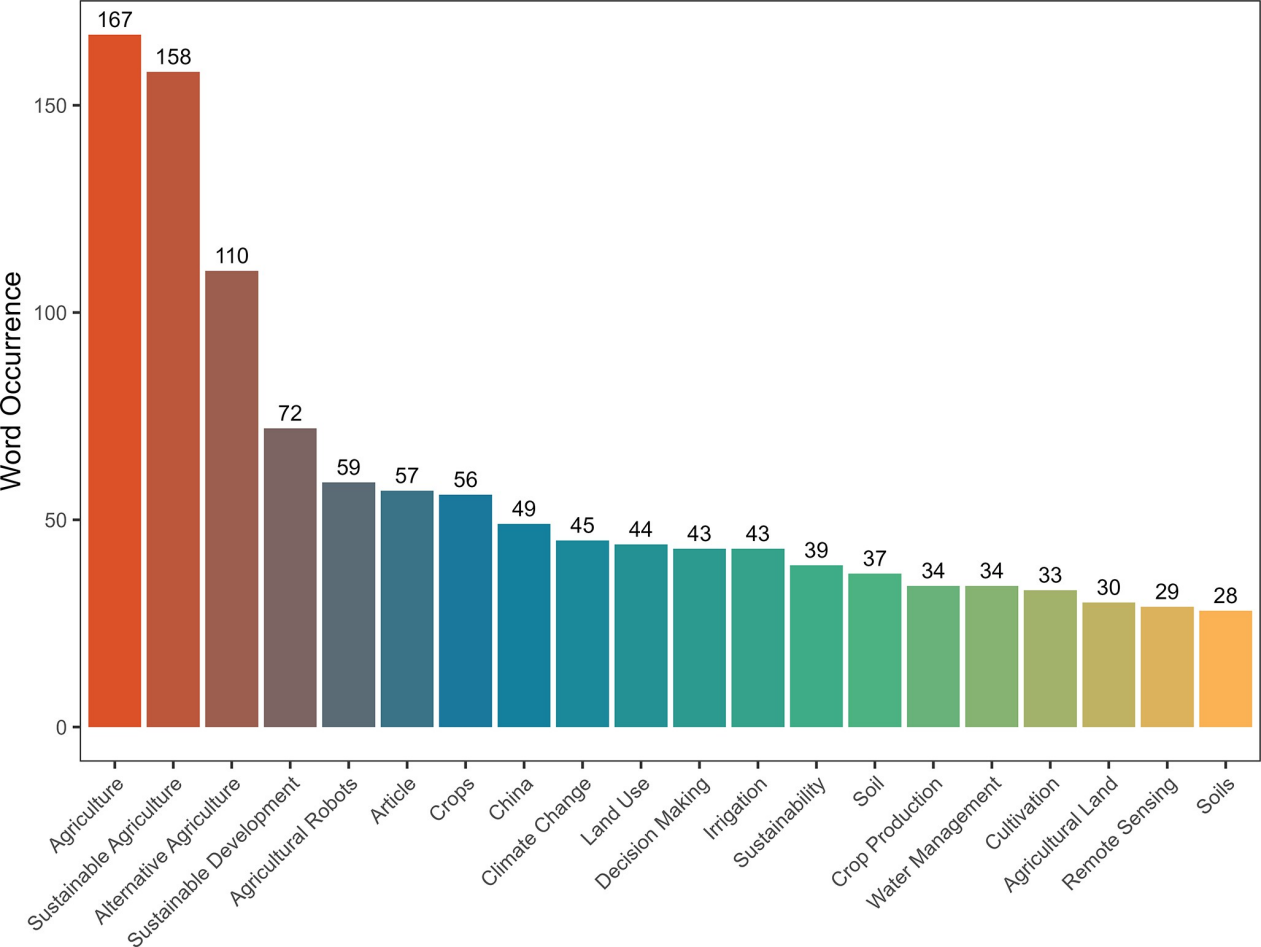
Top keywords in the field of AI and sustainable agriculture.

The result of the keyword analysis using Biblioshiny shows precisely the various technologies within the scope of artificial intelligence which are being used in sustainable agriculture. These technologies are–machine learning, agricultural robots, neural networks, artificial neural networks, remote sensing, precision agriculture and support vectors machines. In addition to it, Fig 3 provided the major keywords and its occurrence in the selected articles. It is evident apart from the keywords of artificial intelligence and agriculture which are under study, the most projected key words are agricultural robots, decision making, machine learning, remote sensing, artificial neural systems, logic algorithms and other related technologies.

The projected set of keywords also helps in determining the various thematic areas in which the majority of the articles belong. The popular thematic areas determined are automation of agricultural production using AI, machine learning in the field of food production, modernization of farming methods using technology and weather forecasting solutions for sustainable agriculture.

### 3.8 Network analysis

**Fig 4:**
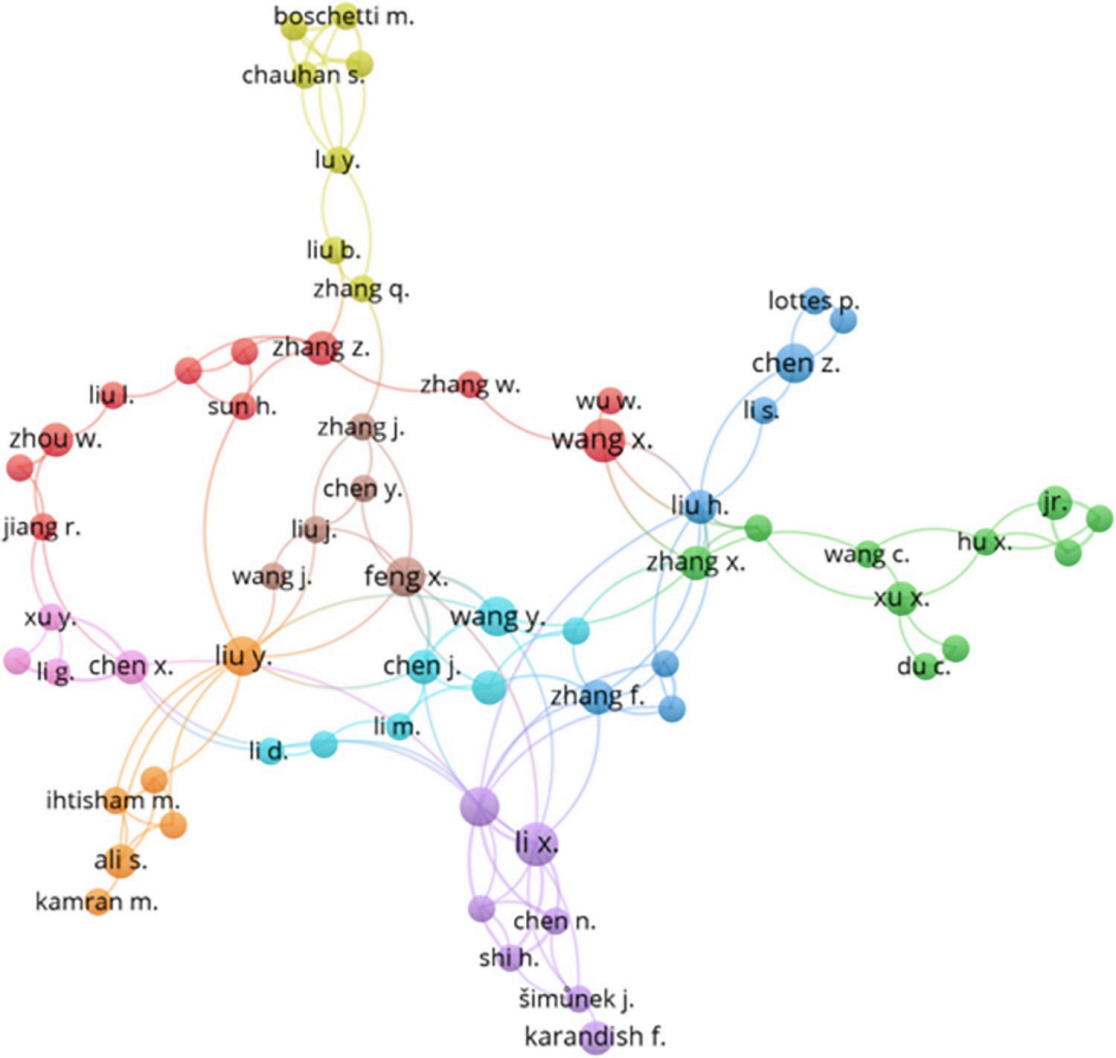
Author collaboration for AI and sustainable agriculture research work.

The Fig 4, is a VOSviewer generated network illustration of the most prominent published author collaborations for AI and Sustainable agriculture scientific articles that are indexed in Scopus from 2000–2021. The eight different color clusters represent the different domains and the interaction and collaboration of authors from different domains to produce multi-disciplinary scientific articles. The size of each circle denoted for each author represents the amount of academic literature, citations they have produced in the field of AI and sustainable agriculture respectively.

**Fig 5:**
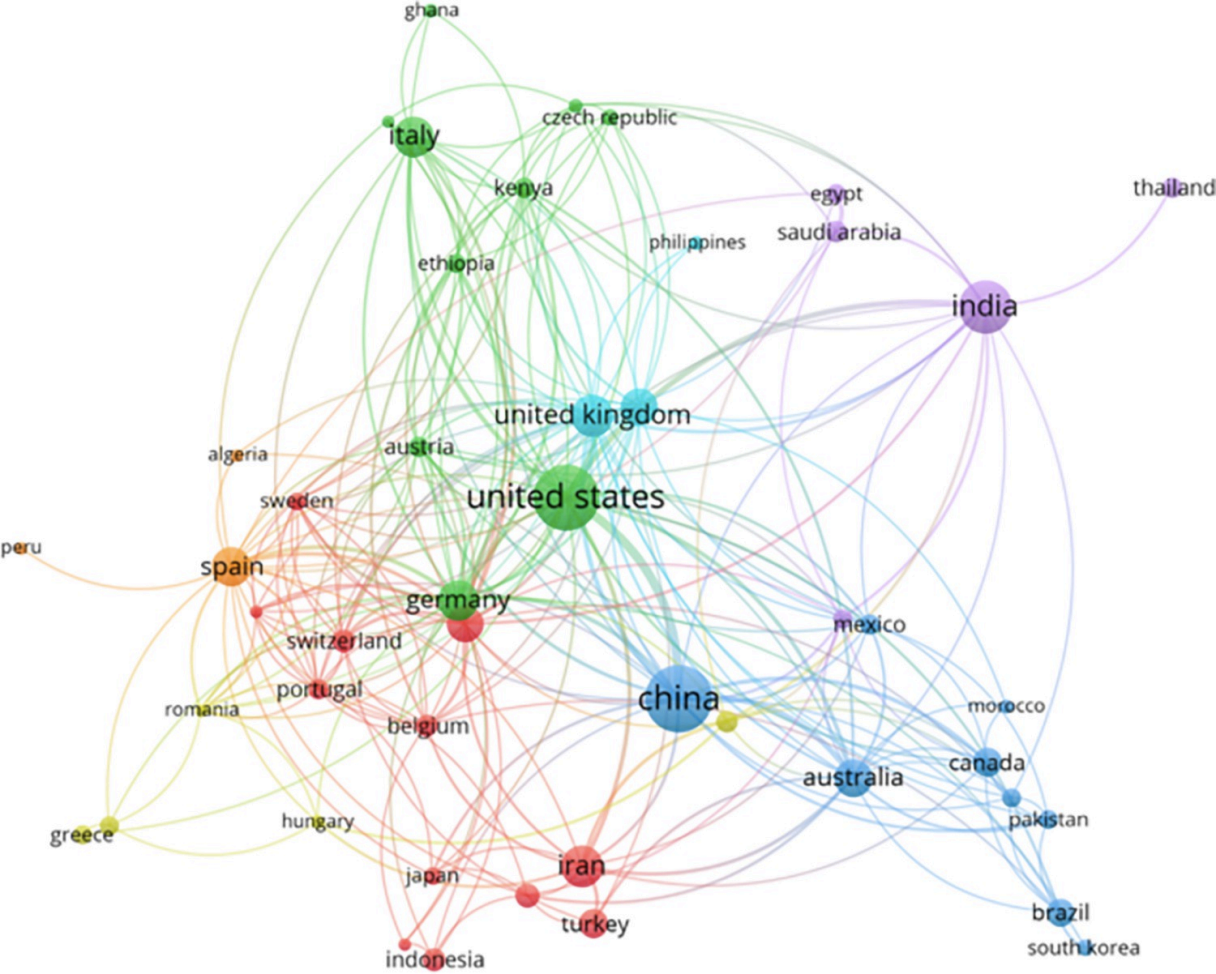
Country collaboration for AI and sustainable agriculture research work.

Based on the originating country of publication and using VOSviewer software the geographic network distribution is obtained in the form of Fig 5. The network also reinforces the findings from the bibliometric analysis of the top 20 countries that produce scientific studies in the domain of AI and sustainable agriculture. The different colored clusters represent the various disciplines of study and their interaction with other disciplines in the scope of the study. Further, the circles representing China, the United States, India, the UK, and Germany confirm the earlier findings. On further exploration of Fig 5, it is observed that the clusters created between the United States of America, European Union member states and United Kingdom are more dense and diverse as compared to the interaction with and amongst Asian countries.

## 4. Discussion

AI enhancements have made different sectors of production highly effective in doing so by providing sustainable solutions. Initially, the applications of AI technologies were intended to increase productivity but the trend had begun to gradually shift towards researching for sustainability and green technologies that would automate and reduce resource consumption in the production process. Jha et al. in their review of automation used in agriculture, highlighted that the younger farmers and producers are more inclined towards investing in automation technologies than the older generations [21]. Over utilization of pesticides and agrochemicals has led previously fertile lands to turn barren, this can be avoided by implementing Artificial Neural Network methods as suggested in the results obtained by Elahi et al. in their 2019 test on rice crops [22]. Studies have also emerged in utilization of data intelligence automation tools like extreme machine learning in precision farming decision support system to determine the accurate yield of crops of small holder farms of produce such as coffee [23, 24].

The aim of this article is to compile and reach various inferences from the bibliometric analysis of the literature published in the duration of 2000–2021. The research is innovative and a novel study trying to explore the AI techniques utilised specifically in sustainable agriculture practices which have not been done before. The resulting research framework is intended to provide a basis for future research in an efficient and organised manner.

### 4.1 AI in sustainable agriculture

The use of AI in sustainable agriculture has the potential to transform aspects of farming such as image sensing for yield mapping, yield prediction, skilled and unskilled workforce, increasing yield and decision-support for farmers and producers [25]. Based on a 2019 article by Alreshidi, AI is widely being implemented in the following ways to make agriculture more sustainable, these are- climate monitoring, automatically climate-controlled greenhouses, crop quality monitoring, livestock management, predictive analysis and comprehensive farm management systems are a few [26]. The AI products that are highly in demand from customers of sustainable agriculture backgrounds are chatbots for help with information on farming practices, digital plant health diagnosis applications, remote sensing instruments and irrigation management solutions [27].

Klyushin & Tymoshenko, (2021) proposed an optimization approach for drip irrigation system optimization to attain sustainable agriculture by using AI methods [28]. On the other hand, Zhang et al. (2021b) emphasized on informatics and material science to find sustainable solutions in sustainable agriculture by using nanotechnology and AI [29]. Aggarwal & Singh (2021) asserted on technology assistance in precision farming and discussed the implications of AI and internet of things (IoT) in agriculture to assess water requirements, humidity, need for fertilizers etc [30]. Spanaki et al. (2021) addressed the issues concerning food security and proposed an AI technique as a solution by adopting a design science methodology [31]. Mohr & Kühl (2021) investigated the barriers in AI acceptance in agriculture and applied technology acceptance model [32]. Nevertheless, Mahto et al. (2021) used artificial neural network (ANN) to forecast prices of agriculture commodities and compared of performance of their model with ARIMA model for sustainable agriculture [33].

### 4.2 Machine learning in sustainable agriculture

Machine learning was defined in the 2018 book “Foundations of Machine Learning” as computational processes that utilize historic data and past experiences to modify, improve, repair and predict future performance accurately [34]. Machine learning in sustainable agriculture latest utilization is in optimizing supply chains [35], in-field monitoring [36], soil temperature prediction [37] and sustainable soil management [38]. The different types of machine learning technologies that can be implemented to foster sustainable production are decision trees, neural networks, polynomial predictive methods and K-nearest neighbors [39]. Traditional methods of soil suitability assessments for sustainable agriculture can prove to be expensive and time taking especially in remote areas where data about the properties of the soil is unavailable there machine learning technologies are gaining popularity for large scale land suitability assessments [40].

Qin et al. in 2018 successfully explored the predictive abilities of machine learning on estimating the economic optimum nitrogen rate for corn crops using data from 47 test conducted throughout the American corn belt in USA and found that more robust data was required to make accurate estimations using machine learning based models [41]. Liakos et al. in 2018 conducted a review of machine learning in agri-tech and found that the artificial and deep neural network method of machine learning was a popular choice across all categories of agriculture processes but specifically in the categories of livestock management, water management and soil management [42]. A 2019 comparative study between four different machine learning techniques by Ju et al., concluded that while conducting estimations on corn and soybean yields, Convolutional Neural Network or CNN was the most accurate [43].

### 4.3 Robotics in sustainable agriculture

Robotics in agriculture is mainly utilized to speed up repetitive and mundane tasks in the production process like spraying, mowing, seeding, harvesting, weed control, picking and finally in sorting products and packaging. Automation provided by robotics in combination with cloud computing, block-chain and big data has also found utility in supply chain of fresh produce. In the realm of making farming sustainable, field robots are used in precision farming by targeted weed control functions replacing treatment of crops and soil with harmful and excessive chemical sprays [44]. The concept of Agriculture 5.0 has been gaining momentum as a term used to define the incorporation of artificial intelligence and robotics in data-driven farming systems [45].

Sarri et al. in 2020 reported the results of SMASH project at its design stages of AgroBot with four modules combined to physically control weeds and protection of crops [46]. Robotic solutions are not only popular in the research community but also amongst the members of the industry who want to invest and implement these sustainable systems [47]. With the widespread utility, there is an emergence in new research interest in safe human-robot interactions in agricultural settings. Benos et al., express that due to automation and programmed robots, human safety is a concern and that efforts must be taken to make robots extra sensitive to perceiving human proximity and ensure risk-free work environment [48].

The article by Linaza et al., from 2021 summarises the recent research projects in European Union on the use of robotics and highlights that the use of robots can not only make it precise but also solve the labour shortages caused rise in the average age of farmers [49]. Furthermore, Mondejar et al., described that usage of robotics in agriculture can solve major food shortage issues and help achieve United Nations sustainable development goals without depleting non-renewable resources rapidly [50, 51]. However, to bring the robotic technologies to the commercial level some barriers that must be overcome are improved speed and accuracy. There is a scarcity of research funding when compared to investment interests in industrial manufacturing and military equipment that’s why the body of study is small and the process from development to implementation is slow and on small scale [52–54].

### 4.4 Resulting observations made in the use of AI techniques in sustainable agriculture

There are a number of other AI solutions that can be implemented in ensuring sustainability in food production like predictive analytics, decision support systems, genomics tracing, artificial neural networks, fuzzy logic, neuro-fuzzy logic, Bayesian Network, and remote sensing. Studies have suggested that advanced bio-sensing technologies in sustainable agriculture will facilitate early diagnosis of diseases and plant pathogens even in asymptomatic plants hence reducing the loss of crops and production [55]. Unmanned Arial Vehicles or drones integrated with advanced machine learning help in continuous weed management enabling selection decisions and reducing herbicide diffusion in the environment [56]. Also, Rasmussen et al., in 2021 studied the effect of engaging unmanned arial vehicles which receive data from satellite imagery to perform weed mapping of a particular variety of weed and the result showed that it provides higher resolution images and makes an individual variety of weed to be detected in the crops [57].

AI and genomics research can enhance defect detection and improve production gains per unit of time with genetic historical data for making accurate predictions [58–60]. Artificial neural networks and fuzzy logic on the other hand are systems that can be continuously trained in making prediction models for sustainable agriculture [21]. A recent 2021 stimulation study by Pazouki, devised a system for surface irrigation using fuzzy expert systems and meta-heuristic optimization algorithms which enhanced the regular system and improved its practicality, performance and reduced the requirement for manual labour [61]. Hence proving the wide application of the system in irrigation and water management for sustainable agriculture.

On the other hand, the application of fuzzy logic was used in another study by Soylu and Çarman in 2021 where they developed an automatic fuzzy-based slip control system for tractors on the field by continuously recording how many times slippage occurred during the process of tilling, which proved to reduce slippage by 42% and reduced fuel consumption by 44% [62]. A few limitations of the artificial intelligence-based systems were highlighted in the recent 2021 study by Meroni et al., in which they stated that the difference between the accuracy of machine learning enabled systems and regular bench-marking systems were not significantly dispersed and the performance of machine learning systems reduced if the data was small [63].

**Fig 6:**
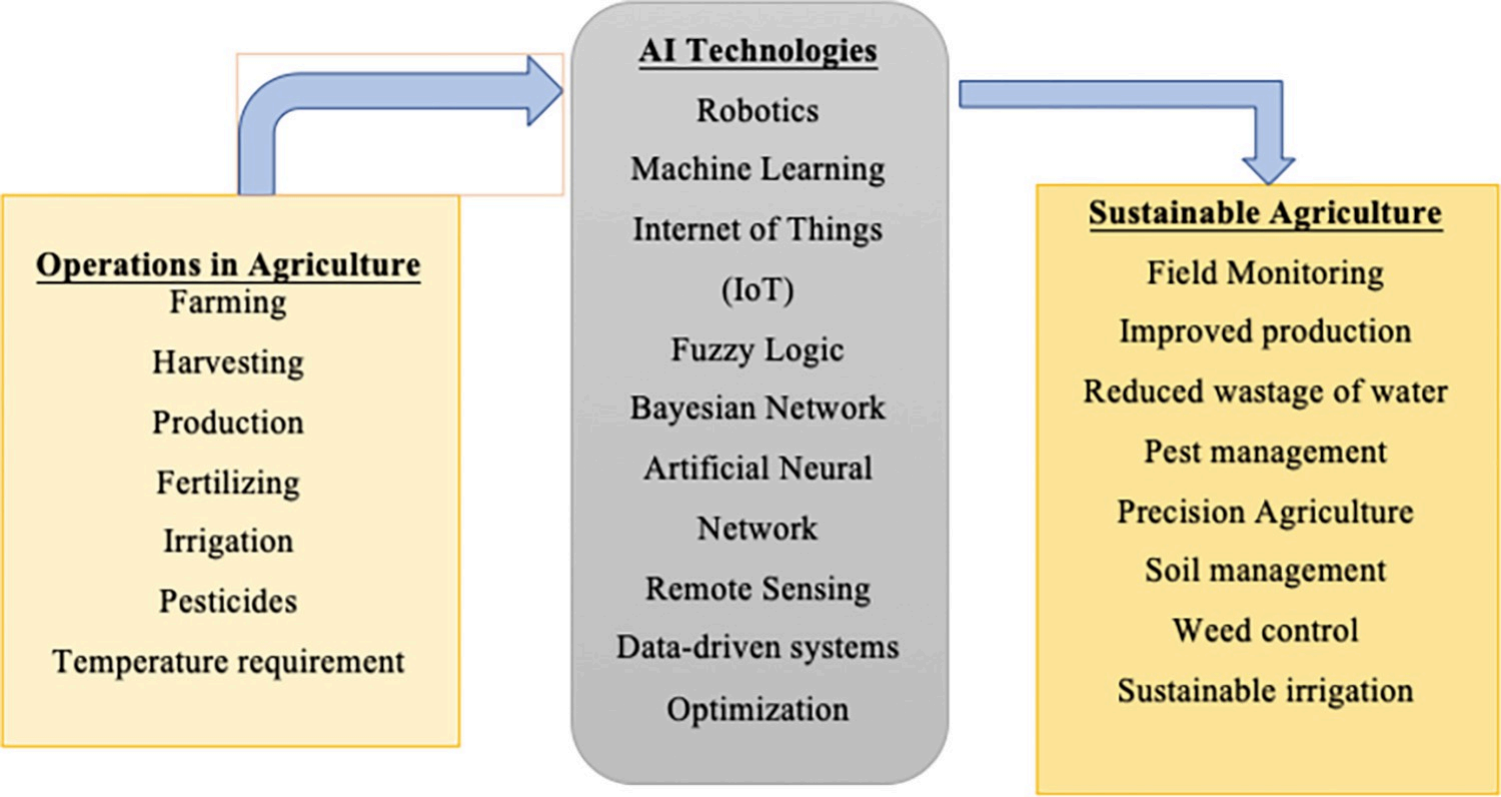
Proposed research framework.

Table 7 briefly discussed some recent studies conducted in the selected field of research. Based on previous literature and identified techniques used in providing solutions for sustainable agriculture, a research framework is developed in this study (Fig 6).

**Table 7:** Some recent studies conducted in the field of AI and sustainable agriculture.

Article	Area	Technique/Application	Objective	Proposition
Knowledge mapping of machine learning approaches applied in agricultural management—A scientometric review with citespace [64]	Agriculture management	Machine learning	To identify recent research based on machine learning methods in agricultural management Presented in a visualised and quantitative format.	Integrated research of more methods in material management.
Citizen science for sustainable agriculture–A systematic literature review [65]	Sustainable agriculture	Citizen science	To identify emerging trends in citizen-science studies	Increase sample size and make research more stakeholder oriented (Ex. Farmers)
A review of applications and communication technologies for internet of things (IoT) and unmanned aerial vehicle (UAV) based sustainable smart farming [66]	Communication technologies, sustainable smart farming	IoT & UAV based sustainable farming	Identify advantages and usages of IoT and UAV in advanced farming methods, IoT, network functions and network essentials for smart farming	Research required in the areas of resource management, hardware maintenance, security issues arising from connected systems, large scale data maintenance
Research advances and applications of bio-sensing technology for the diagnosis of pathogens in sustainable agriculture [55]	Pathogens detection in sustainable agriculture	Bio-sensing	A review of bio-sensor methods for disease identification in food production and the agricultural industry	Further integration of other techniques in increasing sensitivity of autonomous detection bio-sensors
Integrated technologies toward sustainable agriculture supply chains: missing links [67]	Supply chain	Information communication technology	Finding the missing links in the study of utilizing integrated enabling technologies to achieve sustainable, circular agriculture supply chain	Study the technologies enabling further advancement in reaching UN sustainable development goals
Scientometric analysis of the application of artificial intelligence in agriculture [68]	Agriculture	Artificial Intelligence	Scientometric review to identify the academic collection of the application of artificial intelligence in agriculture	Further research specifically focused on precision farming application of artificial intelligence
Automatic identification of diseases in grains crops through computational approaches: A review [69]	Disease identification	Artificial Neural Network	Review of 109 peer-reviewed articles on early stage detection of diseases on maize, rice, wheat, soybean, and barley to improve production. The article provides an integrated taxonomy of grain plant leaf diseases	Additional accurate classification may be improved by integrating optimization processes or techniques based on fuzzy set theory, rough set theory by utilizing classification algorithms in existing literature
A review of autonomous agricultural vehicles (The experience of Hokkaido University) [70]	Agriculture process automation	Robotics	Review of autonomous agricultural vehicles (AAV), their components and their advantages and disadvantages	Continued development of robotic AAV for the benefit of stakeholder in agriculture
A review of remote sensing applications in agriculture for food security: Crop growth and yield, irrigation, and crop losses [71]	Food security	Remote-sensing	Overview of utilization of satellite remote sensing information in analysis and agriculture management in ecohydrology	Development of algorithms that ascertain the yield in heterogeneous agricultural systems
A systematic literature review on machine learning applications for sustainable agriculture supply chain performance [35]	Supply chain performance	Machine learning	Systematic review of 93 research papers on machine learning (ML) solutions in agricultural supply chain process	Use of ML in transforming present production procedures into data-driven smart manufacturing systems and developing customer focused applications based on consumer purchase behaviour

Based on the results and further propositions from the above stated recent studies, we have attempted to propose a framework for optimizing future research in the utilization of AI technology in sustainable agriculture. We propose that, from Fig 6 depicted below, researchers can create novel topics and thematic areas utilizing the classification of operations performed in agriculture at various stages. Then choose relevant and correlatable AI technologies as in Fig 6, that have been identified based on existing academic literature and research its application on the distinct processes identified in attaining sustainable agriculture practices.

The suggested framework can also provide a foundation to attract new researchers to the direction of researching this avenue as it is observed that the size of existing scholarly research is relatively very small between 2000–2021. There is an ethical need to motivate further scholastic interest in the development of modern technology usage in the food supply chain to ensure food safety for future generations owing to rapid depletion of resources and global environmental and climate changes.

## 5. Future research

The potential for future research is vast in the scope of sustainability studies involving advanced technologies and sustainable agriculture as these fields interact frequently in the market’s demand for newer innovations. In terms of bibliometric exploration of previous literary works, further scrutiny could be performed by using sophisticated R bibliometrix programming for functions such as co-occurrence analysis, co-citation analysis, cluster and stream analysis etc. The size of the dataset used for the studies can also be increased to include more technologies such as block chain, Internet of Things, cloud computing, and supply-chain solutions, which are extensively utilized in the agriculture industry. Further studies could also combine scholarly articles and reviews from more than one index source such as Scopus and Web of Science together for extracting a larger dataset. But this kind of dataset will require further steps to clean the dataset from duplications and redundant information.

## 6. Implications

The implications of the results from this study can be of significant impact as it provides the targeted scholars, readers and researchers with a comprehensive compilation of the body of research conducted previously in the area of sustainable agriculture and AI. The results imply that the interest in the exploration of diverse applications of AI in sustainable agriculture is steadily rising. Distinguished authors from multidisciplinary backgrounds and expertise are inclined towards combining it with other thematic domains around the world.

It also indicates that there is potential to publish more literary and research articles as the body of literature are not as large compared to other bibliometric studies. This article also brings the attention of the readers to this untapped opportunity, and they can get motivated to fill this research gap. The proposed research framework in Fig 6, also has the potential to further provide a streamlined approach to building pertinent scholarly research topics in this particular thematic area.

## 7. Conclusion

In our analysis, we classified the keywords and used them to extract 674 relevant articles and reviews which gave us the following results: there is a rising academic interest in the field of AI usage in sustainable agriculture with a drastic improvement from 2019 to 2020. China, the USA and Australia are leaders in producing top works of literature and authors in the domain. These results were obtained by performing data cleansing and classifying the data set for network analysis using functions of Microsoft Excel, VOSviewer, and Biblioshiny. By analysing the results it can be determined that there is huge potential for the application of AI to attain sustainability, especially in predicting the yield, crop protection, climate control, crop genetic control, and produce supply-chain, wherein the prominent researchers and institutions need to collaborate further and form more networks to bring radical progress in the field.

As we proceed to the future, focusing on sustainability will dictate all aspects of life on earth. The first and foremost challenge with climate change will be ensuring food safety and availability for all. Although the green revolution and industrial revolution in the past had exponentially improved food production capacities, these approaches also exerted intolerable pressure on the agricultural lands, natural resources, and the ecosystem, the implications of which are unsustainable and irreversible. The 4th industrial revolution with its hi-tech capabilities promised sustainable alternatives to traditional agriculture practices which can potentially reduce or slow down the depletion of the earth’s resources. As the Covid-19 pandemic shocked and paused the usual shenanigans of every sector for a while, it is a good time to review the body of work in AI and sustainable agriculture before making our way forward.

## Supporting information

S1 Data (CSV)
